# A Gold Nanoclusters Film Supported on Polydopamine for Fluorescent Sensing of Free Bilirubin

**DOI:** 10.3390/s19071726

**Published:** 2019-04-10

**Authors:** Zhou Li, Wenxiang Xiao, Rongen Huang, Yajing Shi, Cheng Fang, Zhencheng Chen

**Affiliations:** 1 School of Life and Environmental Sciences, Guilin University of Electronic Technology, Guilin 541004, China; lz_guet@sina.com (Z.L.); h1615363480@icloud.com (R.H.); 18070764995@163.com (Y.S.); chengfang@guet.edu.cn (C.F.); chenzhcheng@163.com (Z.C.); 2 Guangxi Key Laboratory of Automatic Detection Technology and Instruments, Guilin University of Electronic Technology, Guilin 541004, China

**Keywords:** free bilirubin, polydopamine, gold nanoclusters, fluorescent film

## Abstract

Serum bilirubin is an important biomarker for the diagnosis of various types of liver diseases and blood disorders. A polydopamine/gold nanoclusters composite film was fabricated for the fluorescent sensing of free bilirubin. Bovine serum albumin (BSA)-stabilized gold nanoclusters (AuNCs) were used as probes for biorecognition. The polydopamine film was utilized as an adhesion layer for immobilization of AuNCs. When the composite film was exposed to free bilirubin, due to the complex that was formed between BSA and free bilirubin, the fluorescence intensity of the composite film was gradually weakened as the bilirubin concentration increased. The fluorescence quenching ratio (F_0_/F) was linearly proportional to free bilirubin over the concentration range of 0.8~50 μmol/L with a limit of detection of 0.61 ± 0.12 μmol/L (S/N = 3). The response was quick, the film was recyclable, and common ingredients in human serum did not interfere with the detection of free bilirubin.

## 1. Introduction

Bilirubin is an endogenous compound derived from the breakdown of hemoglobin. Approximately 250~300 mg bilirubin is formed daily in a normal adult. More is formed in the neonate [1]. Bilirubin can be categorized into two forms: conjugated bilirubin and unconjugated bilirubin (or free bilirubin), depending on whether there is glucuronidation or not [2]. After glucuronide conjugation, conjugated bilirubin becomes water-soluble and is nontoxic to tissue. Free bilirubin (fBR) is highly toxic because it can pass through the cell membrane and brain barrier due to its hydrophobicity [3]. Under normal conditions, most of the bilirubin can be metabolized in the liver by conjugating mainly with glucuronic acid, allowing for excretion in the bile [4]. The complex metabolism of bilirubin is disturbed easily by liver dysfunction, leading to high levels of fBR in blood. Excessive fBR has an affinity to nervous tissue and can deposit on brain tissue to cause brain damage or even death [5,6]. Serving as a diagnostic marker of liver and blood disorders, the analysis of fBR is clinically desirable.

As concerns fBR detection in serum samples, various methods can be found in the reported literature. The classical methods are usually based on diazo reactions or vanadate oxidation [7], but the reaction is indirect (needing an accelerator substance) and pH-dependent. Methods using bilirubin oxidase, peroxidase, or an artificial enzyme have been developed for electrochemical detection of fBR [8,9]. The disadvantages of electrochemical fBR biosensors are that they may be susceptible to interference from electroactive species in the sample and the response could be limited to the molecular oxygen in the solution. Some methods using sophisticated instruments have also been applied to improve the selectivity and limit of detection (LOD), such as high-performance liquid chromatography (HPLC) on a reverse-phase (RP) C18 support coupled with thermal lens spectrometric detection [10], liquid chromatography-mass spectrometry (LC-MS) utilizing a molecularly imprinted sol–gel xerogel for bilirubin microextraction [11], and microfluidic chip-capillary electrophoresis [12].

Fluorescence is a powerful tool in the analytical field owing to its simplicity of implementation, high specificity, and high sensitivity [13,14,15]. For bilirubin, no fluorescence in the visible region is observed at room temperature. However, fluorescence appears when a zinc acetate/Tris-DMSO solution is added to a bilirubin solution [16]. Some fluorescent probes have been developed for the sensitive and selective determination of fBR. For instance, Ru(bipy)_3_^2+^ [17] and a yttrium–norfloxacin complex [18] were used as fluorescence probes for fBR based on the quenching effect. A bovine serum albumin (BSA)-stabilized copper nanocluster that was modulated by Fe (III) [19] and mimetic peroxidase [20] was fabricated for bilirubin quantification by fluorescence enhancement. Water-soluble polyfluorenes with a D-glucuronic acid appendage [21], a metal organic framework [22], and a fluorescent new imine [23] were designed for fBR determination utilizing the fluorescence resonance energy transfer (FRET) mechanism.

Most of the abovementioned sensors were developed by dispersing the fluorescent probes in solutions for bilirubin sensing and the probes were difficult to recycle. Apparently, these one-off sensors may increase the reagent consumption, experimental cost, and risk of environmental pollution. Thus, developing a surface sensor is necessary to overcome these disadvantages. Gold nanoclusters (AuNCs) are a new kind of fluorescent nanomaterial with the advantages of a long lifetime, a large Stokes shift, and biocompatibility. AuNCs have found application in cell imaging, enzyme mimics, biosensors, and photovoltaics [24,25,26,27,28] Gold nanoclusters have been studied extensively in solution. However, only a few studies have discussed the production of an optical AuNCs film. Chen’s group reported a recyclable fluorescent AuNCs membrane for copper (II) ion sensing by using the excellent membrane-forming ability of BSA under the isoelectric point [29]. The thick film (ca. 100 μm) resulted in a relatively long response time (10 min). AuNCs were also embedded into polymeric hosts for optical measurements [30,31]. For a sensing purpose, physical entrapment by a polymer would produce a thick film and suffer from leaching of the AuNCs.

In this work, we prepared a gold nanoclusters film supported on polydopamine (PDA) for fluorescent sensing of fBR. BSA-templated AuNCs were covalently bonded onto polydopamine film. BSA acted as the biorecognition element for fBR and AuNCs as the fluorescence reporting unit. The film showed a good response towards fBR by fluorescence quenching.

## 2. Materials and Methods

### 2.1. Reagents and Instrumentations

Bovine serum albumin (BSA) and chloroauric acid (HAuCl_4_.3H_2_O) were purchased from Sigma-Aldrich and Shanghai Reagent (Shanghai, China), respectively. Glutaraldehyde, bilirubin, and all other reagents used in the experiment were obtained from Aladdin Reagent (Shanghai, China). Bilirubin (1 mg) was first dissolved with 0.1 M NaOH (0.1 mL) and then diluted with 50 mM phosphate-buffered solution (PBS) (pH 7.4) to 10 mL to obtain a stock solution (171 μM). Chemicals and solvents were all of analytical reagent grade unless otherwise stated. Ultrapure water (18.2 MΩ, Milli-Q, Millipore) was used throughout the experiment.

Fluorescence spectra and UV-vis absorption spectra were measured with a Hitachi F-4600 spectrofluorophotometer and a Hitachi UH-5300 spectrophotometer, respectively. Scanning electron microscopy (SEM) images were obtained from a field emission SEM (Hitachi S-4800). The morphology and size of the gold nanoclusters were characterized by a JEOL 2100 high-resolution transmission electron microscope (HRTEM) operating at an accelerating voltage of 200 kV.

### 2.2. Preparation of the Gold Nanoclusters Film by Polydopamine Adhesion

AuNCs were synthesized according to [32]. All glassware used in the preparation of AuNCs should be cleaned first with aqua regia and then rinsed thoroughly with ultrapure water. A solution of HAuCl_4_ (20 mL, 10 mM, 37 °C) was added to BSA solution (20 mL, 50 mg/mL, 37 °C) under vigorous stirring. After 2 min, NaOH solution (1 M) was introduced until pH = 12, and the mixture was incubated at 37 °C for 8 h. The solution color changed from light-yellow to light-brown and finally to deep brown. The obtained AuNCs were further purified by dialysis (MWCO 35 kDa) in ultrapure water for 24 h to remove excessive reactants. The final solution was lyophilized and stored at 4 °C for further use.

Dopamine was dissolved in 10 mM Tris-HCl (pH 8.5). A quartz glass chip was cleaned sequentially with acetone, ethanol, and water under ultrasonication. Then, the chip was vertically dipped into dopamine solution (2 mg/mL) under mild stirring at room temperature for 12 h. Through the self-polymerization of dopamine, polydopamine particles were formed and coated onto the substrate. The coated surface was rinsed with distilled water and dried under a stream of nitrogen. The chip was dipped in 2% concentration of glutaraldehyde for 2 h, washed subsequently in water, and dried with nitrogen. Finally, the composite film was obtained by incubating polydopamine with 50 mg/mL AuNCs in PBS buffer (50 mM, pH 7.4) in a moisture chamber at room temperature for 8 h. After incubation, the chip was rinsed with PBS to remove the unreacted AuNCs, dried in air, and stored at 4 °C for use.

### 2.3. Fluorescence Measurements

The chip with the PDA/AuNCs film was mounted with a solid sample holder and placed in a 10 mm quartz cuvette. The cuvette was positioned in the fluorimeter to ensure excitation and emission at 45 degrees to the surface. An edge filter (long wave pass, 500 nm) was used before the emission monochromator to cut off the scattered light from excitation. The split width for the excitation and emission monochromators was set at 5 nm. Different concentrations of bilirubin solutions (2 mL) were added to the cuvette and equilibrated with the film for 2 min. The fluorescence emission intensity was recorded at 597 nm with an excitation wavelength of 487 nm at 25 ± 1 °C.

## 3. Results and Discussion

### 3.1. Characterization of PDA/AuNCs Film

**Figure 1:**
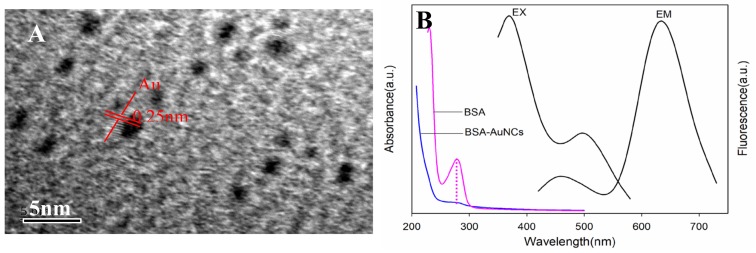
HRTEM images of gold nanoclusters (AuNCs) (**A**) and their absorption and fluorescence spectra (**B**).

Gold nanoclusters were prepared by using BSA as a reducing and protecting agent. The solution was dark brown and emitted intense red fluorescence under ultraviolet (UV) light (365 nm). As depicted in the HRTEM image, AuNCs were spherical particles with an average diameter of ca. 1 nm. The lattice fringes of AuNCs had a discerned lattice spacing of 0.25 nm, which was ascribed to the d spacing of the {111} crystal plane of metallic Au (Figure 1A). The lattice spacing value is consistent with the previous report [33].

The absorption and fluorescence spectra of AuNCs are presented in Figure 1B. The BSA-AuNCs had a weak peak centered at 280 nm, which belonged to the absorption of BSA (Figure 1B). For the AuNCs solution, there were two excitation peaks centered at 370 nm and 495 nm, respectively. When excited at 370 nm, the BSA-AuNCs displayed dual emission peaks at 440 nm and 638 nm. The weak blue peak came from BSA surface oxidation species and the strong red emission arose from the core AuNCs unit.

**Figure 2:**
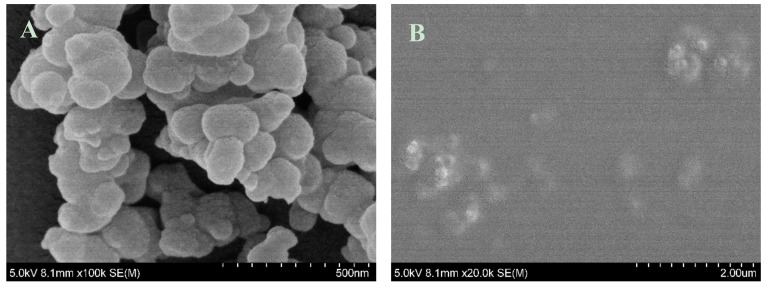
SEM images of the PDA film (**A**) and PDA/AuNCs film (**B**).

The PDA film was prepared by the self-polymerization of dopamine in alkali media. SEM was carried out to characterize the morphology of the PDA film before and after AuNCs immobilization (Figure 2). As shown in Figure 2A, polydopamine nanoparticles with diameter of about 100 nm were formed spontaneously onto the quartz glass. Polydopamine film was used as an adhesion layer to immobilize the BSA-AuNCs for bilirubin sensor fabrication. The film surface became smooth after BSA-AuNCs immobilization (Figure 2B).

**Figure 3:**
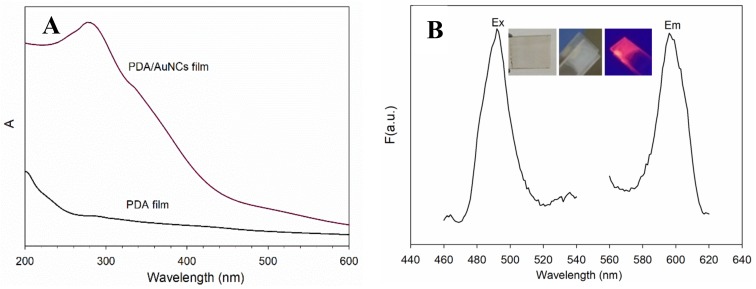
UV-Vis spectra (**A**) and fluorescence spectra (**B**) of AuNCs/PDA film (Ex: excitation, Em: Emission). Inset: images of the PDA film (left), the PDA/AuNCs film (middle) under visible light, and the AuNCs/PDA film under UV light (365 nm) (right).

The UV-Vis spectra of the PDA and PDA/AuNCs film are shown in Figure 3. There was no obvious absorption peak for the PDA film. Once BSA-AuNCs were bonded onto PDA, a broad peak centered at 280 nm was observed, which was attributed to BSA absorption. The PDA/AuNCs film was transparent under visible light and emitted strong red fluorescence under UV light (365 nm) (Figure 3A). The fluorescence spectra of the film are presented in Figure 3B. No obvious fluorescence band of polydopamine was observed. After BSA-AuNCs were immobilized, the film emitted the typical red emission of AuNCs. Compared to BSA-AuNCs solution, the excitation at 370 nm and the emission at 440 nm of the film disappeared. Moreover, the wavelengths of excitation and emission were blue-shifted to 487 nm and 597 nm, respectively. The blue shift may be ascribed to the binding between BSA-AuNCs and the solid PDA film, which restricted the molecular motion, and the intercluster distance became very short. The cluster−cluster interactions changed the energy transfer between the surface ligands of neighboring clusters and increased the energy spacing between the ground state and the excited state, resulting in a blue-shift emission [30].

The fluorescence of the AuNCs/PDA film could be modulated by changing the concentration of AuNCs solution for binding. Concentrations of 20, 30, 40, 50, and 60 mg/mL AuNCs solutions were investigated for film fabrication, and the fluorescence intensities were 30 ± 5, 53 ± 8, 78 ± 12, 257 ± 14, and 464 ± 24, respectively. It is obvious that a higher concentration of AuNCs for immobilization is favorable for improving the fluorescent performance of the film.

### 3.2. PDA/AuNCs Film for Fluorescent Sensing of fBR

It is known that fBR is transported in blood by binding strongly with serum albumin to form a water-soluble complex before liver metabolism, and this binding lowers the level of fBR in blood plasma [1]. Based on the interaction between fBR and the BSA template of AuNCs, a PDA/AuNCs composite film was designed for the sensitive and selective determination of fBR. In the sensing film, BSA and AuNCs acted, respectively, as the recognition component and the signal reporting unit for bilirubin detection. It was found that fBR could significantly quench the fluorescence emission of the sensing film due to the formation of a non-fluorescent complex. Therefore, by using the PDA/AuNCs composite film as a sensing platform, a method was established for fluorescent determination of fBR in serum.

**Figure 4:**
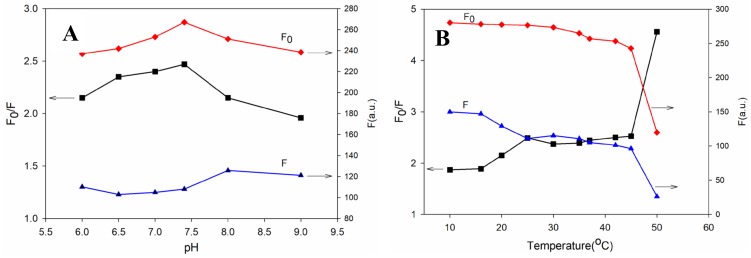
The effect of pH (T = 25 °C) (**A**) and temperature (pH = 7.4) (**B**) on the fluorescence response of the AuNCs-PDA film to bilirubin (50 μM).

Fluorescence may be influenced by pH and temperature. Hence, their effect on the fluorescence response of the film to bilirubin was investigated by adjusting the temperature and pH value of PBS solution (50 mM) (Figure 4). Figure 4A illustrates the value of F_0_, F, and F_0_/F (where F_0_ and F are the fluorescence intensities for the sensing film in the absence and presence of 50 μM bilirubin, respectively) of the film at different pH values. The change in pH value exerted a moderate influence on F_0_ and F. The relatively low fluorescence quenching F_0_/F of the film in pH < 7.4 media might be ascribed to the decrease in bilirubin solubility at a lower pH value. However, higher pH values could destroy the formation of the bilirubin–BSA complex to reduce fluorescence quenching. Physiological pH (7.4) was suitable for bilirubin detection. The effect of temperature on bilirubin sensing is shown in Figure 4B. A higher quenching efficiency was achieved beyond physiological temperature, while the BSA-AuNCs exhibited a drop in emission intensity upon raising the temperature. Hence, bilirubin determination could be carried out at 25 °C.

**Figure 5:**
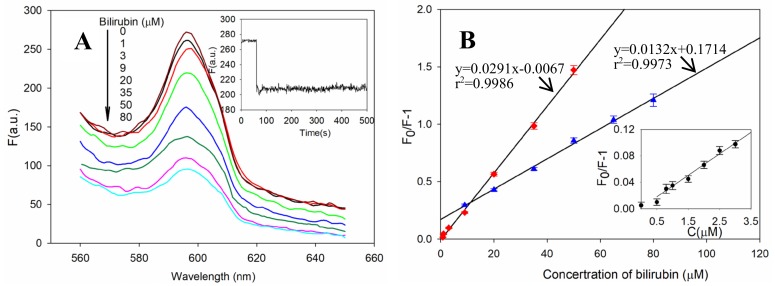
(**A**) Fluorescence quenching of the PDA/AuNCs film caused by increasing concentrations of bilirubin. Inset is the dynamic fluorescence quenching over time upon bilirubin (10 μM) addition. (**B**) The calibration curve for bilirubin sensing when using 50 mg/ml (square) and 60 mg/ml (triangle) of AuNCs for immobilization. Three repeat measurements were done for each set of data. Inset displays the linear curve in the concentration region of 0–3 μM bilirubin.

When the PDA/AuNCs film was exposed to bilirubin, the fluorescence of AuNCs was quenched notably due to the formation of a non-fluorescent BSA–bilirubin complex. The interaction equilibrated within 2 min. The fluorescence intensities of the film at 597 nm decreased gradually as the bilirubin concentration was increased (with an excitation wavelength of 487 nm) (Figure 5A).

The fluorescence of the PDA/AuNCs film was gradually quenched by increasing the concentration of bilirubin (Figure 5A). The calibration curve was established by using the PBS solution at pH 7.40 and 25 °C. The sensing ability of the film could be adjusted by changing the concentration of AuNCs for immobilization. Using a lower concentration of AuNCs (≤50 mg/ml), the film had higher bilirubin sensitivity than those with a higher concentration of AuNCs (≥60mg/ml). A wider linear range could be achieved when the film was prepared in a higher concentration of AuNCs, as shown in Figure 5B. Taking sensitivity and linear range into account, 50 mg/ml of AuNCs was suitable for film preparation. The linear curve of the film was achieved in the concentration range of 0.8~50 μM. The calibration equation was expressed as: F_0_/F−1=0.0291C (μM)−0.0067, with an LOD of 0.61 ± 0.12 μM of bilirubin (S/N = 3). The relative standard deviation for the five replicate measurements of 10 μM was 2.3%.

Santhosh et al. reported a fluorometric probe based on human serum albumin (HSA)-stabilized AuNCs for fBR detection in homogenous solution, which had a linear range of 1~50 μM and an LOD of 0.246 μM (S/N = 3) [34]. The main differences between our work and Santhosh et al.’s are as follows: (1) AuNCs were bound covalently onto the PDA film by surface chemistry. The film sensor could be recycled and was integrated easily with the device, avoiding an increase in reagent consumption and experimental cost. (2) Covalent immobilization of AuNCs helped to improve the sensitivity. In this film sensor, BSA acted as the biorecognition unit for fBR. Although the binding of BSA with bilirubin is 2–13 times weaker than that of HSA, this sensor achieves a comparable sensitivity and linear range to the reported literature [34]. Moreover, BSA is cheaper than HSA. (3) AuNCs in the surface binding state had distinct fluorescence properties. Being different from the situation of probes in solution or polymer solids, the molecular motion of BSA-AuNCs on PDA/AuNCs film is inhibited completely [30,35]. Thus, the energy spacing might be altered, resulting in blue-shift fluorescence both for excitation and emission. On the other hand, surface immobilization would change the distribution of probes and the microenvironment of the BSA matrix, which may exert influence on the sensing properties of the film [36].

**Figure 6:**
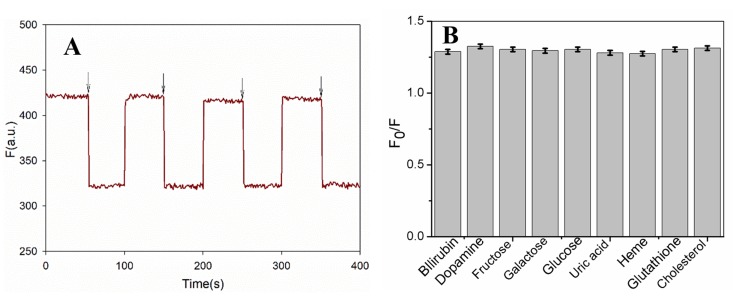
The recyclability of the AuNCs-PDA film by repeated exposure to bilirubin and NaOH (**A**) and the effect of coexisting substances on free bilirubin (fBR) sensing (**B**).

Reusability is an important parameter for sensors. After interacting with bilirubin, the fluorescence of the PDA/AuNCs film could be regenerated by 0.1 M NaOH and then washing with pure water. The film sensor was interacted repeatedly with 10 μM of bilirubin and 0.1 M of NaOH to determine the reusability. The quenching efficiencies (F_0_/F) were measured and are shown in Figure 6A. There is no significant change in the fluorescence response of the film towards bilirubin in each sensing/regeneration cycle, indicating that the sensor has acceptable reusability.

An interference study was carried out to investigate the specificity of this method for bilirubin detection. The effect of some common substances present in blood serum, such as heme, dopamine, galactose, glucose, fructose, uric acid, glutathione, and cholesterol (1000 μM each), was investigated by having them coexist with 10 μM of bilirubin. The experimental results revealed that the coexisting compounds did not interfere with bilirubin detection (Figure 6B).

### 3.3. Determination of fBR in Human Blood Serum

To evaluate the feasibility of the film, sensing studies were carried out for detecting fBR in human serum. Human blood serum was obtained from No.181 Hospital in Guilin City, China. Unknown concentrations of bilirubin were determined by the standard addition method. Then, some known concentrations of bilirubin were spiked with human serum samples and the obtained results are listed in Table 1. The proposed methods have good recoveries, in the range of 98–105%, which suggests that the developed biosensor has the potential to detect bilirubin in human serum.

## 4. Conclusions

In order to overcome the disadvantages of one-off sensors that are dispersed in solutions during analysis, a recyclable sensor was developed for the detection of fBR based on a PDA/AuNCs film. PDA was deposited on a quartz slide and BSA-AuNCs were covalently bonded onto the film through glutaraldehyde linking. Surface immobilization of the fluorescent probes provided a quick and sensitive response towards fBR. The sensor is suitable for the determination of fBR in human serum.

## Figures and Tables

**Table 1:** The determination of fBR in human blood serum samples.

Sample	Determined (μM)	Spiked (μM)	Found ^a^ (μM)	RSD ^b^ (%)	Recovery ^c^ (%)
Sample 1	5.73 ± 0.34	5.00	10.96 ± 0.28	2.6	104.6
		20.00	25.65 ± 1.10	4.3	99.6
Sample 2	7.85 ± 0.45	5.00	12.92 ± 0.31	2.4	101.4
		20.00	27.88 ± 1.31	4.7	100.2
Sample 3	15.87 ± 1.08	5.00	20.78 ± 0.66	3.2	98.2
		20.00	36.02 ± 1.30	3.6	100.6

^a^ Value = mean ± SD (*n* = 3). ^b^ RSD = relative standard deviation. ^c^ Recovery = (Found-Determined) × 100%/Spiked.
